# Optimized production of a safe and efficient gene therapeutic vaccine versus HIV via a linear covalently closed DNA minivector

**DOI:** 10.1186/1471-2334-14-S2-P74

**Published:** 2014-05-23

**Authors:** Roderick Slavcev, Chi Hong Sum, Nafiseh Nafissi

**Affiliations:** 1 School of Pharmacy, University of Waterloo, Waterloo, Canada

## Introduction

Conventional plasmid vectors (pDNA) possess unwanted prokaryotic DNA that when delivered to the mammalian cells can reduce transgene expression and integrate into the human genome, potentiating oncogenesis. Bacterial sequence-free, linear covalently closed (LCC) DNA minivectors have shown improved safety and efficacy both in vitro and in vivo. We previously constructed and characterized an in vivo LCC DNA minivector (ministring) system that can convert pDNA into DNA ministrings. Here we sought to optimize this system and apply ministrings to encode a gene to generate HIV virus-like particles (VLPs) in targeted cells. The DNA-VLP expresses gagV3(BCE) gene in target cells to simultaneously yield GagV3(BCE) VLP assembly and presentation of immunodominant HIV antigens, eliciting production of neutralizing antibodies and cytotoxic T-cell responses against HIV.

## Methods

A one-step invivo linear covalently closed (LCC) mini-plasmid production system that conditionally expresses the tel protelomerase was constructed in Ecoli (R-cells) to enable efficient production of DNA ministrings from a specialized pDNA. LCC gagV3(BCE) DNA ministrings were produced from the constructed precursor pGagV3(BCE) parent plasmid (from Dr. Chil-Yong Kang) when passaged through R-cells. Protease gene deletions were introduced into R-cells to sustain protelomerase expression while limiting the detrimental effects of heat shock-induced recombinant protein degradation upon prolonged heat induction. Plasmid and DNA ministring derivatives were lipoplexed with a RGD-complexed gemini surfactant and transfected into immortalized leukemia K562 cells with induced surface expression of CD51/CD61, and assessed for cell trafficking, transfection efficiency and viability.

## Results

R-cells with a hflX protease-associated gene deletion significantly imcreased DNA gagV3(BCE) DNA ministring yields to greater than 90%, compared to ~70% in wildtype cells. DNA ministrings indicated remarkably improved cellular trafficking and nuclear translocation of the vector, significantly higher transfection rates and LCC DNA proved far safer than the circular pDNA, where cells having undergone unwanted vector integration events into were targeted for cell death.

## Conclusion

We optimized a robust technology conferring one-step in vivo production of an efficient and safe DNA ministring gagV3(BCE) DNA-VLP vaccine gene therapeutic versus HIV.

